# The Complex but Fascinating Relationship between Sport and Atrial Fibrillation: From Pathophysiology to the Clinical Scenario

**DOI:** 10.3390/jcdd10060255

**Published:** 2023-06-11

**Authors:** Mario Tatangelo, Marco Rebecchi, Marianna Sgueglia, Alessandra Colella, Cinzia Crescenzi, Germana Panattoni, Pellegrino Ciampi, Oreste Lanza, Emanuele Canali, Leonardo Calò

**Affiliations:** 1 Division of Cardiology, Policlinico Casilino, Via Casilina 1049, 00169 Rome, Italy; 2 BIND Department, University of Palermo, Piazza Marina, 61, 90133 Palermo, Italy

**Keywords:** atrial fibrillation, athletes, arrhythmia, endurance sport, cardiac remodeling

## Abstract

Atrial fibrillation (AF) is the most common cause of hospital admission among all arrhythmias in the general population. Moreover, AF represents the most common arrhythmia in the athletic population as well. The complex but fascinating relationship between sport and atrial fibrillation has not yet been fully clarified. Although the benefits of moderate physical activity in controlling cardiovascular risk factors and in reducing the risk of atrial fibrillation have been widely demonstrated, some concerns have been raised about the potential adverse effects of physical activity. Endurance activity in middle-aged men athletes appears to increase the risk of AF. Several different physiopathological mechanisms may explain the increased risk of AF in endurance athletes, including the imbalance of the autonomic nervous system, changes in left atrial size and function and presence of atrial fibrosis. The goal of this article is to review the epidemiology, pathophysiology and clinical management for AF in athletes, including pharmacological and electrophysiological strategies.

## 1. Introduction

Atrial fibrillation (AF) is the most common cause of hospital admission among all arrhythmias. In most cases, it does not lead to sudden death and has traditionally been considered a benign arrhythmia. However, recent evidence suggests that AF could be responsible for a significant reduction in the quality of life and an overall increase in mortality, especially in patients affected by structural heart diseases [1]. The AF clinical approach should therefore be directed not only towards the prevention of acute short-term complications but also towards ensuring the normal duration of and quality of life. This goal conflicts with the difficulty of implementing therapies that are able to restore and maintain normal cardiac activity. As an estimation, just over a third of people with AF achieve effective and lasting control of their heart rhythm [2]. The restoration of normal sinus rhythms, as well as maintaining this and preventing further episodes, is a challenge for modern cardiology, which may involve different strategies, including combination therapies. Atrial fibrillation is the most common arrhythmia in the athletic population and is more common in middle-aged than in younger athletes [3]. Although the benefits of moderate physical activity in controlling cardiovascular risk factors and in reducing the risk of atrial fibrillation have been widely demonstrated, some concerns have been raised about the potential adverse effects of vigorous physical activity, particularly regarding the risk of arrhythmias in endurance athletes. Several different physio-pathological mechanisms may explain the increased risk of arrhythmia in athletes. Cardiac adaptation to intense exercise results in increased vagal tone, lower resting heart rate and increased systolic output, ventricular dilation, and hypertrophy, all of which may cause a predisposition to AF recurrences [4].

## 2. Objective

AF is the most common tachyarrhythmia in the general population and in athletes, and it may impair athletic performances and decrease athletes’ quality of life. Numerous factors may influence athletes’ vulnerability to AF, including autonomic nervous system changes, inflammation, fibrosis and functional parameters. It is important to assess whether the risk of the development of AF is higher in athletes compared with the general population. We noticed a lack of consistent data in the current literature; therefore, our aim is to provide an overview of available knowledge about the topic with a narrative review that may be a stimulus for further research.

## 3. Methods

### 3.1. Search Strategy

We searched the PubMed database for all available studies reporting the relationship between athletic physical training and atrial fibrillation. The search had a language limitation with only articles written in English included, and only research published before December 2022 was considered. The following keywords were used: “atrial fibrillation”, “athlete”, “sport”, “physical training”, “inflammation” and “autonomic nervous system” alone and in various combinations, using key Boolean terms. Abstracts were reviewed, AF-related articles regarding the sports population were reviewed in full and a complete analysis of the bibliography of the selected articles was performed. References of the retrieved articles and the review articles published by expert authors on the subject were also screened for eligible studies.

### 3.2. Study Eligibility

Any type of study reporting the above-mentioned relationship between athletic physical training and atrial fibrillation were considered eligible for inclusion. Studies reporting AF in the general nonathletic population were excluded.

## 4. Epidemiology of Atrial Fibrillation in Athletes

In recent years, several epidemiological investigations regarding AF have been performed according to age, years of training and, finally, the type of sport practiced. The first article, suggesting an increased risk of atrial fibrillation in healthy middle-aged men practicing sports, was published by Karjalainen et al. [5] in 1998. The authors studied 300 high-level veteran athletes compared to 495 controls. All individuals enrolled were between the ages of 35 and 59 and were retrospectively assessed using a questionnaire after 11 years. The incidence of AF was evaluated after excluding from both groups all individuals who had ended their sporting activities, had died or had developed risk factors for AF during the follow-up. In this study, AF was observed in 5.3% of athletes compared to 0.9% of controls (*p* = 0.012) with an AF annual incidence of 0.48 and 0.08%, respectively. Although the authors concluded that sporting activities were related to an increase in AF risk in healthy middle-aged men, the study presented some limitations, but it was an important starting point for reflection on the topic and stimulated further studies. In fact, Mont et al. [6], in a retrospective analysis of 1160 consecutive patients affected by lone AF (LAF), showed that among men with LAF, the percentage of those who practiced regular sports was higher than in general population (63 vs. 15%). The same population was analyzed in a case–control study with two age-corresponding controls from the general population for each case [7]. The analysis showed that sports practice increased the risk of developing LAF by more than five times [odds ratio, OR 5.6 (1.35–19)]. In 2006, Heidbuchel et al. [8] observed, among middle-aged patients who had undergone a typical atrial flutter ablation, a higher AF incidence in those engaged in endurance sports when compared with athletes who practiced non-resistance sports (81 vs. 48%, *p* = 0.02, respectively). This study suggests that a history of endurance sports participation may increase the development of AF after ablation for atrial flutter. Although these former studies have shown a possible association between exercise and AF, other investigations have not suggested an increased risk of atrial fibrillation in professional athletes. In 2005, Pelliccia et al. [9] found an AF prevalence of 0.3% among 1777 healthy athletes (age: 24 ± 6 years); this was therefore similar to that observed in the general population, with no difference between those with a left atrial diameter smaller than 40 mm or greater than 40 mm. The scientific relevance of these findings has to be related to the large population of athletes engaged in different sports, despite the limitations represented by a relatively young overall population and the left atrial size measurement, only limited to the anterior–posterior diameter. Moreover, Abdulla and Nielsen [10], in a different population of athletes, showed that the risk of AF developing was significantly higher than in non-athletes or in the general population (OR (95% confidence interval, IC = 5.29 (3.57–7.85); *p* = 0.0001 and Z-score = 8.08). Despite this, the available sample of studies was small, and controls were not adequately matched by age in all studies. In addition, the results could also be associated with several biases attributed to the variation in endurance sports practiced by the different types of athletes across the studies. Recently, Newman et al. [11], in their meta-analysis, showed a higher relative AF incidence in a younger population of athletes under 55 years (odds ratio, OR 3.6 (2.08–6.29)) than in those over 55 years (odds ratio, OR 1.76 (0.96–3.21)). Despite this, the latter presented a relative AF incidence higher than the non-athletes and should therefore not be ignored. As a further consideration, AF in athletes is not necessarily associated with an increased risk of death. This possibly occurs because of other factors, such as a low prevalence of coronary heart disease in this population, can reduce the overall risk of mortality, in addition to the evidence of persistent atrial fibrillation that develops only in a minority of male endurance athletes. Age seems to be an important factor in the type of AF in athletes, as Hoogsteen et al. reports [12]. In younger athletes, the first arrhythmic attack was adrenergically induced, with a typical onset in daytime and was provoked by exercise, stress or stimulants (e.g., caffeine); however, in contrast, in older athletes it was vagally induced, as paroxysmal AF appeared at night, in the immediate hours after intensive exercise or after heavy meals. The average participant ages in different investigations (Table 1) were varied and the samples frequently included middle-aged athletes. The potential influence that age may have on the type of AF could be a stimulus for further research.

## 5. The Borderline between Beneficial Physical Activity and the Risk of Atrial Fibrillation: From Endurance to Mixed Activity

The beneficial role of physical activity in terms of reducing cardiovascular disease and all-cause mortality has been clear for several years and is recommended by current guidelines [13]. However, it is unclear whether all cardiovascular conditions, including AF, can benefit from physical activity. Their relationship is not fully clarified. The risk of developing AF seems to be related to the type and intensity of exercise. Endurance sports, including cycling, cross-country skiing, running and orienteering, have been shown to be associated with an increased risk of AF [10]. On the other hand, there is no satisfactory evidence of an AF excess in non-endurance athletes. A possible explanation, at least in part, may be that endurance sports often require much higher levels of training and physical conditioning and, therefore, greater hemodynamic stress over a longer time [14]. A recent review by Elliot et al. [15] documented a reduction in AF incidence in athletes who practiced aerobic sports of greater than 8 METS when compared to endurance sports participants. After a complete adjustment for known risk factors, they verified a significant reduction (30%) in AF risk for third quartile activity participants (mean aerobic activity: 9.3 MET) but not for those in upper quartile (average aerobic activity: 11.6 MET). Moreover, the Henry Ford Exercise Testing project [16] involved 64,561 adults (46% women) followed for an average of 5 years. Those patients underwent stress testing, and the incidence of AF was documented by reviewing medical reports. In this study, high levels of activity were associated with a 56% reduction in AF risk after adjustment for multiple risk factors. For each MET achieved during treadmill testing, the AF risk was reduced by up to 7%. The potential mechanisms behind the reduction in atrial fibrillation incidence with aerobic physical activity are still unclear. The CARDIO-FIT study [17] showed that patients with significant improvements in aerobic capacity also had lower blood pressure, lower inflammatory status and better glycemic control, in addition to lower body weight, suggesting an overall improvement in risk factors. There were also notable improvements in both left ventricular (LV) size and diastolic function, and a significant reduction in left atrial (LA) size. When these improvements were achieved together, benefits for patients with AF were substantial. Based on the evidence presented so far, it is reasonable to conclude that maintaining an active and fit lifestyle reduces the risk of AF. However, these benefits do not seem to extend to those practicing endurance exercise far beyond the recommended volume in current guidelines [18]. In the Physician’s Health Study [19] nearly 17,000 men were studied with an average of 12 years follow-up: the authors noticed a 20% increase in AF risk among athletes who trained vigorously for 5–7 days a week. A systematic review [20] and a meta-analysis [10] in this regard suggested that athletes who were engaged in long-term endurance workouts displayed an increased incidence of atrial fibrillation. As previously mentioned, an increased AF risk has been observed in studies regarding different sizes, types of sports, ages and exercise modalities. There is an increasing need for investigations that are able to identify the maximum and safe regular “dose” and the most appropriate type of sport before the risk of AF developing becomes significant. Despite much current evidence suggesting a potential increase in the risk of AF with endurance exercise, a recent meta-analysis found a significant association between the type of exercise and the risk of developing AF, with mixed sports conferring a greater risk than endurance sports [11]. The relationship between mixed sports and an increased risk of AF is difficult to interpret because of the wide range of sports analyzed, while the true effects of specific training models further complicate the process. In fact, training volume may be an important risk factor for the development of AF and deserves further research. 

KEY POINTS

- An active and fit lifestyle reduces the AF risk.
- The risk of developing AF seems to be related to the type and intensity of exercise.
- Endurance exercise far beyond the recommended volume in current guidelines seems to be associated with higher AF risk.

## 6. Sport and Atrial Fibrillation: Pathophysiology

**Figure 1:**
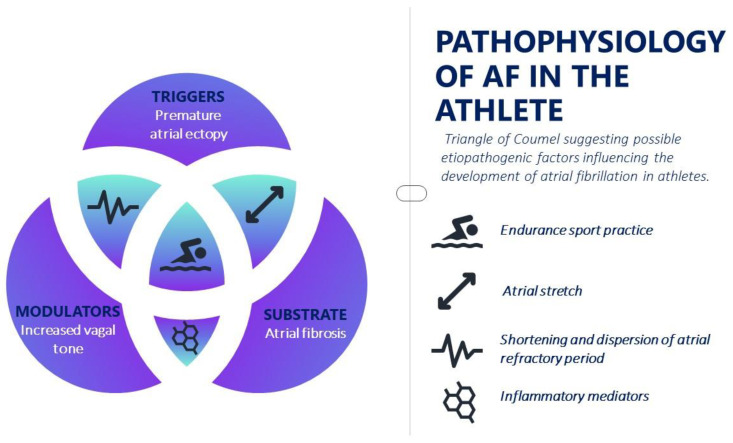
Pathophysiology of atrial fibrillation in the athlete.

Although the physiopathology scenario of AF is complex, the initiation and maintenance of this arrhythmia is commonly due to a focus that determines rapid supraventricular extrasystoles or through re-entry circuits triggered by ectopic foci, either as a result of late post-depolarization or after early post-depolarization. Today, Coumel’s triangle [21] still represents the clearest method of understanding the complex pathophysiological AF mechanism, identifying three important elements: electrical and structural remodeling, triggers and the role of the autonomic nervous system (Figure 1). Structural and electrical changes within the atrium that lead to a reduction in atrial refractoriness and therefore to conduction slowdown and electrical dispersion are known to facilitate the formation of re-entry circuits and atrial fibrillation as a result.

### 6.1. Autonomic Nervous System Changes

AF persistence is further facilitated by changes in the autonomic nervous system that are able to exacerbate an ectopic focus (sympathetic and parasympathetic activation) or shorten the atrial refractory period (parasympathetic activation). Although few studies have evaluated atrial ectopic beat activity in athletes, there is some scientific evidence that the extra-systolic burden may be higher in athletes than in controls. In runners with a longer training history, there was a small but significant increase in premature atrial contractions compared with those performing fewer hours of training [22]. Similarly, the frequency of premature atrial contractions was higher in professional athletes than in amateur athletes or in a group of sedentary controls matched for age and sex [23]. However, in both studies, the overall premature atrial contraction burden was low compared to that observed in AF patients, possibly indicating that ectopic atrial activity, at least at rest, may not always play a significant role in inducing AF in athletes. Moreover, in both animal and human models, continuous physical training determines an imbalance of the autonomic nervous system (ANS), resulting in a greater parasympathetic activation and a reduction in the sympathetic tone [4]. Although such changes are thought to be cardioprotective, they may increase atrial susceptibility to arrhythmia, mainly as a result of a shorter atrial refractory period dependent on potassium currents mediated by acetylcholine release. It has also been shown that abrupt changes in the sympathovagal balance, often observed at the start of exercise and during recovery, may precede AF onset. Although both parasympathetic and sympathetic tone shorten atrial refractory time, more heterogeneous atrial parasympathetic innervation produces greater arrhythmic susceptibility [24]. Endurance exercise results in increased vagal activity which could facilitate AF inducibility. In an experimental model in rats undergoing the endurance exercise equivalent to 10 years of training, vagal activity was increased as expected, predisposing them to AF, but rapidly decreased after a period of detraining [4]. In contrast, another subsequent study in mice undergoing physical exercise showed that the inhibition of inflammatory mediators, such as TNF-alpha, reduced exercise-induced negative remodeling, resulting in a decreased AF inducibility, but had no effect on positive remodeling, including increased vagal tone. These data suggest that factors other than the ANS also contribute to the pathogenesis of sports-induced AF [25].

### 6.2. Inflammation and Oxidative Stress

Inflammation plays an important role in promoting and maintaining atrial fibrillation, involving both structural and electrophysiological alterations that lead to atrial remodeling. In endurance athletes, it is well known that strenuous and continuous exercise may promote a generalized pro-inflammatory status, depending on the quantity and quality of exercise not followed by appropriate rest. The inflammatory state is mediated by cytokines such as IL-6, IL-8, and TNF-α; after a mechanical or biochemical myocardial insult, those pro-inflammatory cytokines promote the recruitment of immune cells that infiltrate the atrial myocardium. Monocytes, Th2, and mastocytes, in the late stage of inflammation, promote tissue fibrosis by secreting profibrotic factors such as TGFβ and enhance the activation and differentiation of fibroblasts in myofibroblasts [26]. In addition, profibrotic factors induce augmented ROS (reactive oxygen species) production through mitochondria, ending in the activation of p38 and ERK1/2 pathways, enhancing the transcription of fibrotic genes. ROS are normally produced by mitochondria through oxygen metabolism, but an excessive increase may directly affect ion channels and the propagation of action potential [27].

### 6.3. Atrial Enlargement and Fibrosis

Structural remodeling, in terms of left atrial enlargement, seems to predispose an individual to AF and this occurs in endurance sports as an adaptation to exercise. The increase in atrial size is probably determined by the hemodynamic stress of high-intensity exercise, where maximum cardiac output often reaches 30 L/min. During exercise, both right atrial pressure and pulmonary capillary pressure are markedly high, and it is likely that over time, with prolonged exercise, this leads to ventricular enlargement. A study based on echocardiographic two-dimensional speckle tracking (STE) aimed to characterize myocardial deformation in master athletes with a history of paroxysmal AF, has shown that elite athletes have significant differences in the left atrial function when compared with controls. In fact, a normal left atrial reservoir function but a reduced left atrial contribution to left ventricular diastolic filling was observed. This phenomenon seems to be associated with normal and even super-normal diastolic function and is accompanied by the shift of the left ventricular filling period towards early diastole, mainly due to an increase in the flexibility, elasticity and distensibility of the left ventricular myocardium. In addition, veteran male athletes had significantly higher LA volumes than non-athletes. In contrast, STE-LA values were similar in athletes and non-athletes with paroxysmal AF and significantly lower than in those without AF, suggesting that LA functional parameters are more closely related to AF than volumetric parameters in veteran athletes [28]. In addition to atrial enlargement, the presence of fibrosis within the atria is part of the arrhythmic substrate for the development of AF. In humans, the presence of fibrosis or areas of low voltage are significant predictors of recurrent AF in patients undergoing transcatheter ablation. The development of fibrosis after endurance exercise was demonstrated in a mouse training model, where 16 weeks of training resulted in a significant increase in the right and left atrial fibrosis. The development of fibrosis in conjunction with vagal hypertonus led to the increased susceptibility to atrial fibrillation. Curiously, it is not the complete reduction in atrial fibrosis but the reduced inducibility of AF that determines a detraining period, suggesting that atrial fibrosis is a key factor but not the only mechanism contributing to the risk of developing AF [25]. 

### 6.4. BMI and Diet

Lifestyle factors, including diet, weight and BMI, play a role in AF pathogenesis. Weight and BMI are strictly connected with AF onset, its persistence and even its recurrence after ablation [29]. BMI and progression from paroxysmal to permanent AF have a direct dose–response relationship in the study by Tsang et al. [30]. Obesity seems to be an independent risk factor regarding the pro-inflammatory status associated with the condition, and robust data support this hypothesis. Jamaly et al. [31] provide data regarding the impact of dramatic surgical weight loss on incident AF, and they found that every 5-unit BMI increment raised the risk of incident AF by 33%. On the other hand, in the same study weight loss was independently associated with a reduction in AF risk of 31%. The LEGACY trial [32] supports the importance of weight loss in AF patients as well: after a rigorous weight loss program, there were higher rates of arrhythmia-free survival. Weight loss appears to be crucial to the point that the scientific community is starting to consider the possible role of surgical weight loss in AF populations. In this light, to be more conservative, diet interventions could be assumed to be of relevance; however, despite much conjecture, little evidence exists to advocate for a specific dietary approach [33]. The Mediterranean diet supplemented with extra virgin olive oil resulted in a lower incidence of AF in PREDIMED study [34], but more data are needed to confirm Mediterranean diet superiority. Independent of the dietary approach chosen, a high dietary sodium intake is associated with the long-term risk of new-onset atrial fibrillation and should be minimized [35]. Alcohol is a well-known risk factor for AF and is estimated that 5% to 10% of all AF cases are caused by alcohol consumption [36]; emblematic of this is the “Holiday Heart Syndrome”, in which AF onset in structurally normal heart is associated with alcohol abuse. Direct cardiotoxic effects, including inflammation in myocardial cells, are produced by alcohol abuse and upregulated sympathetic tone, which can be one of arrhythmia’s triggers. 

### 6.5. Exogen Stimulant Agents and Supplements

Stimulant agents, such as guarana, taurine, tea and energy drinks in general, may play a role in AF pathogenesis in terms of their effects on ANS, and some cases of AF have been described in the context of the overconsumption of energy drinks. Athletes might consume a large quantity of energy drinks and/or consume various types of supplements during their sports activity or in the peri-work out period. The American Food and Drug Administration and the European EMA do not fully regulate energy drinks/supplements; there might be certain unreported ingredients, which, in addition to caffeine or alcohol, could enhance the risk of arrhythmias. Despite its stimulant effects, the role of caffeine alone is different. Several trials, including cohort, case–control and observational studies and meta-analyses, have shown no significant association between habitual caffeine consumption and an increased risk of AF. Habitual caffeine drinkers instead display cardioprotective effects due to the blocking of transforming growth factor signaling and LA refractory period lengthening, decreasing the likelihood of arrhythmia development [37]. Among athletes’ supplements, creatine deserves a special mention, a substance utilized by athletes to enhance exercise performance and help muscular growth. In a case report [38] and other previous anecdotal reports linking creatine to the development of arrhythmia, creatine monohydrate was found to be a probable cause of the atrial fibrillation in an otherwise healthy 30-year-old Caucasian man. Athletes and in general people at risk for AF should be cautious of supplement consumption as resulting effects are still uncertain.

KEY POINTS

- Imbalance and changes in ANS may promote AF onset and persistence.
- Strenuous and continuous exercise seems to cause a generalized pro-inflammatory status, which may promote and maintain AF.
- Atrial enlargement and fibrosis are part of the arrhythmic substrate for the development of AF.
- Diet, BMI and body weight act in AF pathogenesis and prognosis.
- Supplements should be consumed carefully in AF and athlete populations.

## 7. Sport and Atrial Fibrillation: Clinical Management 

**Figure 2:**
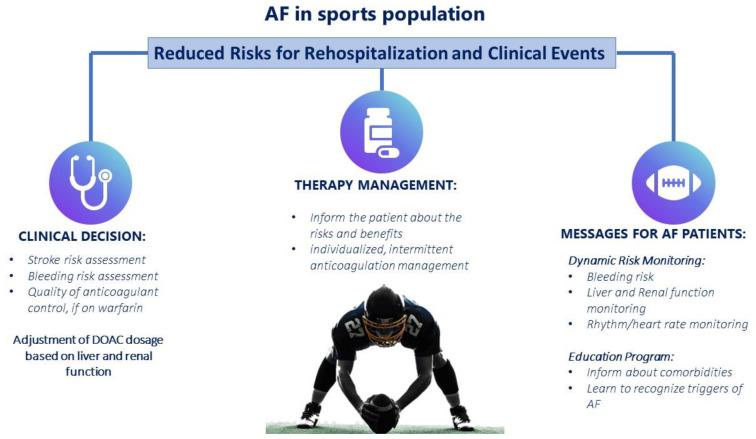
Clinical management of atrial fibrillation in the sports population.

The same principles of symptom reduction and stroke prevention that guide the management of AF in the general non-sporting population also apply to athletes (Figure 2). However, there are several factors unique to athletes that can influence their management. The clinical scenarios varies from asymptomatic patients to acute fatigue and exercise intolerance with the onset of AF. As in non-athletes, the reason for the heterogeneity in symptomatology is intriguing but remains unexplained. Certainly, the identification of overtraining in athletes is important, as the reduction in or temporary cessation of exercise may reduce or even prevent the recurrence of AF. The initial approach should be the recommendation of a reduction in physical activity. The role of exercise in promoting AF should be discussed with the athlete as well. Endurance sports are known to increase the risk of AF, and it is reasonable to assume that reducing physical activity may reduce the frequency and burden of AF. However, there is limited evidence to support this strategy and many athletes may choose to accept the small theoretical risks associated with continued participation [12]. Importantly, complete detraining is discouraged, as a relatively sedentary lifestyle was even more strongly associated with AF prevalence [17]. Moreover, it is also important to rule out hyperthyroidism, alcohol abuse and (illicit) drug use. In symptomatic athletes, the target of pharmacological therapy is represented by rhythm and rate control (in association to rhythm control), with a heart rate of less than 80 bpm, is often necessary. In asymptomatic AF, rate control <110 bpm is reasonable as long as the patient remains asymptomatic and left ventricular systolic function is preserved. Therapeutic rate control is effective in the general population but is difficult to achieve in athletes; beta-blockers are often poorly tolerated by athletes, in part because of their effect on exercise capacity, and non-dihydropyridine calcium channel blockers are not potent enough to slow the heart rate during exertional AF. Moreover, in elite athletes, beta blockers are banned in certain types of sports unless the athlete is able to obtain a TUE (therapeutic use exemption) from the World Anti-Doping Agency. As previously stated, in athletes, rhythm control is generally preferable to heart rate control. Flecainide and propafenone (class Ic) are effective for paroxysmal AF and acute cardioversion (pill-in-pocket approach) in athletes with structurally normal hearts [39]. Side effects include atrial flutter and atrial tachycardia with rapid ventricular response. Disopyramide (class Ia) is effective in vagal and bradycardia-dependent AF but is now rarely used because of its strong anticholinergic and proarrhythmic effects. Amiodarone (class III) is one of the most effective antiarrhythmic drugs for the treatment of AF, but it has long-term toxic effects such as pulmonary and hepatic toxicity, and it is therefore not recommended in younger, healthier populations, including athletes. Athletes with persistent symptomatic AF who have failed or are intolerant to medical therapy should be referred for AF ablation. Despite the cardiac remodeling that occurs in athletes, AF ablation has been shown to be effective in athletes as in non-athletes [40]. After ablation, 77% of athletes were able to return to their prior activity without any restrictions, even though new data show lower rates of athletes who are free from atrial arrythmias compared with non-athletes at 24 months post-ablation and beyond. This difference could be explained by overstimulation in athletes and/or a different underlying substrate for AF. Furthermore, a higher rate of recurrent atypical atrial flutter after AF ablation was reported [41]. As previously shown, it seems prudent to advise athletes to consider a stepwise approach, starting with a period of detraining—often disagreed with by the athlete—to determine whether AF is associated with exercise or not. Prior CHA2DS2VASC score and the need for anticoagulant therapy after ablation are other aspects that need to be evaluated, particularly in those athletes practicing contact sports. An early invasive approach with catheter ablation might be reasonable not only in those who quickly prove intolerant to medical therapy but also in selected athletes who elect ablation as a first-line strategy: recent data show ablation as an acceptable initial rhythm control strategy, at least for symptomatic AF athletes [42,43]. An athlete’s preference has to be considered in a complex shared decision-making process. 

According to current guidelines, if there is no recurrence of atrial fibrillation within 1 month after a successful ablation procedure, the patient can return to sports [44]. The same strategy used in the general population for stroke prophylaxis with anticoagulants should be used in athletes. The prescription of direct oral anticoagulants (DOAC) depends on the clinical risk profile (mainly CHA2DS2-VASc score). Sports involving direct physical contact or sports prone to trauma should be avoided in patients on DOAC [44]. However, with the newer oral anticoagulants, a short-term withdrawal strategy can be devised for some athletes, allowing them to return to full sporting activity. Several studies, based on the pharmacokinetics/pharmacodynamics of DOAC in athletes treated for deep vein thrombosis, have proposed interesting strategies for DOAC discontinuation in relation to the sports competition timing. The rationale of these methods is to encourage sporting activity at a time when the lowest DOAC concentration is reached after intake. This strategy is able to minimize competition-related hemorrhagic risk and, at the same time, is able to reduce thrombotic risk by shifting the time of anticoagulant resumption to the end of a competition, when the risk of hemorrhagic trauma is practically absent [45,46]. 

KEY POINTS

- Athletes with AF can present with heterogeneous clinical symptoms or being asymptomatic.
- Reduction in physical activity should be an initial approach in AF athletes.
- Symptomatic athletes should undergo medical therapy or be referred for ablation.
- DOAC, when needed, can be considered in AF athletes alongside different strategies.

## 8. Conclusions

Based on current evidence, moderate physical activity according to recommended guidelines may not be sufficient to reduce the incidence of AF. On the contrary, endurance exercise clearly increases the risk of AF in men, a condition associated with significant morbidity and mortality. However, two important observations should be emphasized. First, the prevalence of AF in endurance athletes appears to be relatively low, affecting <3% of athletes. Second, resistance exercise confers a mortality benefit that persists despite an increased risk of AF, possibly due to a significant reduction in the risk of other cardiovascular diseases, such as coronary heart disease. Thus, the data concerning the risk of AF due to resistance exercise should be strongly contrasted with data showing a significant benefit. However, it is clear that resistance exercise promotes atrial fibrillation in a small minority of otherwise healthy athletes. The overall conclusion regarding AF and exercise is that any arrhythmic event in an athlete must be assessed through a precise, individualized analysis. Although the term AF is a generic one, it can cover different underlying pathogenetic mechanisms, requiring different therapeutic approaches. Athletes with chronic AF requiring treatment with a DOAC according to clinical risk, can benefit from the personalized timing of therapy to allow participation in training/competitions, particularly those engaged in contact or traumatic sports.

## Figures and Tables

**Table 1:** Epidemiology of atrial fibrillation in athletes.

Authors, Year, and Country	Study Design	Number of Athletes	Age	Years and Type of Training	Main Conclusions
Karjalainen (1998), Finland [5]	Case-control study	300	35–59	11 Long term vigorous exercise	AF risk increased in veterans
Mont (2002), Spain [6]	Case-control study	1160	<65	21 ± 12 All types	Higher LAF in sportsmen
Hoogsteen (2004), Netherlands [12]	Observational study	30	48.1 ± 7.8	>10 (mean 16.5 ± 9.3) Running and cycling	AF changed into permanent in a minority of well-trained athletes
Elosua (2005), Spain [7]	Case–control study	70	<65	No sport practice/Former sport practice <1500 h/life or sport practice >1500 h/life All types	Cumulated lifetime and current sport practice is associated with LAF increased risk
Pelliccia (2005), Italy [9]	Case-control study	1777	24 ± 6	from 2 to 28 (mean 6) All types (elite athletes)	AF prevalence <1% in elite athletes as in general population
Heidbuchel (2006), Belgium [8]	Case-control study	137	53 ± 9	Not specified Endurance sports	A history of endurance sports activity is associated with the development of AF after ablation of atrial flutter
Abdulla (2009), Denmark [10]	Meta-analysis	655	51 ± 9	Various Mixed and endurance sports	AF risk is significantly higher in athletes compared with not athletes
Newman (2021), UK [11]	Meta-analysis	6816	39–72	Various Mixed and endurance sports	AF risk greater in athletes, in particular in those participating in mixed sport and younger athletes

## Data Availability

Not applicable.
